# “I am accustomed to something in my body causing pain”: a qualitative study of knee replacement non-improvers’ stories of previous painful and stressful experiences

**DOI:** 10.1186/s12891-023-06423-9

**Published:** 2023-04-18

**Authors:** Vibeke Bull Sellevold, Unni Olsen, Maren Falch Lindberg, Simen A. Steindal, Arild Aamodt, Anners Lerdal, Alfhild Dihle

**Affiliations:** 1 Lovisenberg Diaconal University College, Lovisenberggata 15B, Oslo, 0456 Norway; 2 Faculty of Health Sciences, Department of Nursing and Health Promotion, Oslo Metropolitan University, Oslo, Norway; 3 Department of Public Health Science, Institute of Health and Society, Faculty of Medicine, University of Oslo, Oslo, Norway; 4 Department of Orthopaedic Surgery, Lovisenberg Diaconal Hospital, Oslo, Norway; 5 Institute of Nursing, Faculty of Health Studies, VID Specialized University, Oslo, Norway; 6 Department of Interdisciplinary Health Sciences, Institute of Health and Society, Faculty of Medicine, University of Oslo, Oslo, Norway; 7 Department of research, Lovisenberg Diaconal Hospital, Oslo, Norway

**Keywords:** Interviews, Pain stories, Persistent postsurgical pain, Total knee arthroplasty, Qualitative research.

## Abstract

**Background:**

Approximately 20% of total knee arthroplasty patients experience persistent postsurgical pain one year after surgery. No qualitative studies have explored previous stories of painful or stressful life experiences in patients experiencing persistent postsurgical pain after total knee replacement. This study aimed to explore stories of previous painful or stressful experiences in life in a cohort of patients that reported no improvement in pain one year after total knee arthroplasty.

**Methods:**

The study employed an explorative-descriptive qualitative design. Data was collected through semi-structured interviews five to seven years after surgery, with patients who reported no improvement in pain-related interference with walking 12 months after total knee replacement. The data was analyzed using qualitative content analysis.

**Results:**

The sample consisted of 13 women and 10 men with a median age of 67 years at the time of surgery. Prior to surgery, six reported having at least one chronic illness and 16 reported having two or more painful sites. Two main themes were identified in the data analysis: Painful years - the burden of living with long lasting pain, and the burden of living with psychological distress.

**Conclusions:**

The participants had severe longlasting knee pain as well as longlasting pain in other locations, in addition to experiences of psychologically stressful life events before surgery. Health personnel needs to address the experience and perception of pain and psychological struggles, and how it influences patients’ everyday life including sleeping routines, work- and family life as well as to identify possible vulnerability for persistent postsurgical pain. Identifying and assessing the challenges enables personalized care and support, such as advice on pain management, cognitive support, guided rehabilitation, and coping strategies both pre-and post-surgery.

## Background

Patients with advanced knee osteoarthritis (OA) are considered eligible for total knee arthroplasty (TKA) if they have pain and reduced function of the knee that are not relieved with other treatments [1]. However, there are conflicting guidelines on when to recommend knee replacement and the decision should also be based on individual and patient-specific factors [2]. Substantial growth in the number of TKA procedures over the next decade is expected [3, 4]. However, approximately 20% of TKA patients experience persistent postsurgical pain (PPP) one year after surgery, with 15–19% reporting severe pain [5–7]. Some patients with PPP one year after surgery have been shown to have a prolonged pain recovery after surgery achieving satisfactory pain levels five to seven years after surgery [8].

PPP is defined as pain that develops or increases in intensity after a surgical procedure, lasts for at least 3–6 months and significantly affects health-related quality of life (HRQOL) [7]. A considerable body of quantitative research suggests that longlasting preoperative pain, pain catastrophizing and pain in anatomical sites other than the knee are the strongest predictors of PPP [9–11]. In a real-life setting, these risk factors may indicate that patients with PPP following TKA have struggled with other painful conditions, with pain coping or have had a more complex history of pain than those who do not experience PPP. Pain and symptoms that are unrelated to the knee may also persist following TKA and may interfere with rehabilitation. For example, in a recent study, patients struggling with higher pain and symptom burden, often unrelated to the knee, were more likely to be non-improvers after TKA [12].

With the high and increasing number of TKA surgeries being performed, targeting, exploring, and gaining more qualitative insight into risk factors for developing PPP are warranted. A previous qualitative study explored the path leading to hip and knee arthroplasty. The patients’ quality of life (QOL) deteriorated as their knee pain increased, until the point where it was experienced as “unbearable” [13]. Another qualitative study which explored the decision-making process leading to TKA surgery, showed that the participants’ description of their knee pain varied from just a nuisance to excruciating pain [14]. However, to our knowledge, no qualitative studies have explored stories of previous painful or stressful life experiences in patients experiencing PPP after TKA. Stories could provide insights into the lived life behind the numbers, representing the patient perspective, as “Language, words, and stories are the currency of the humanities—they are fundamental to the human experience” [15]. With their stories, the participants can share their experiences and we can learn from the stories and make sense of their world [15]. Stories communicate in a way that includes both the particpants as well the reader [16]. Therefor, the stories invites the readers into what is experienced as important real life issues and struggles for a group of non-improvers after TKA surgery. Through stories, nurses, physicians and physiotherapists may better connect and relate to the experiences these patients had earlier in life [16]. Studies have shown that healthcare professionals have limited knowledge of their patients as persons, including previous stories of painful and stressful events in life. The lack of insight into the patient´s life experiences can jeopardize tailoring patient-centered care [17–19]. TKA patients’ previous painful conditions and stressful experiences may prove to be important for preoperative counseling and for designing a more tailored rehabilitation to improve outcomes. Therefor, this study aimed to explore stories of previous painful or stressful life experiences in a qualitative study of a cohort of patients reporting no improvement in pain one year after TKA [5, 8].

## Methods

### Study design

This study employed an explorative-descriptive qualitative design [20] and data was collected using semi-structured individual interviews. This design is suitable when there is little knowledge on a phenomenon, such as the experiences of painful or stressful events earlier in life for patients with PPP. Individual interviews allow for a more focused understanding of each patient’s experiences and permit each participant to elaborate on their own stories without interruptions. The interviewer may also pose follow-up questions when needed [20]. This paper is reported according to the Consolidated criteria for reporting qualitative research (COREQ) checklist [21].

### Participants

This follow-up study focuses on a subgroup of 45 (22%) of 202 patients who participated in a previous longitudinal study of pain, symptoms and HRQOL, and reported no improvement of pain-related interference with walking 12 months after TKA [5, 8, 22]. Patients were invited in the longitudinal study if they were ≥18 years, literate in Norwegian, scheduled for primary TKA for OA and had no diagnosis of dementia. Patients undergoing unicompartmental or revision surgery were not included [5]. We recruited a purposive sample [23] of participants from this non-improver subgroup, and those who attended their five-year follow-up and lived within a two-hour drive from the hospital were eligible for inclusion in this study (n = 31).

### Data collection

Two of the authors (AD, VBS) conducted individual semi-structured interviews at one timepoint, between February 2018 and August 2020. The senior qualitative researcher (AD) trained the first author (VBS) in the interview approach. The first author (VBS) observed while the senior researcher (AD) interviewed the first participants. Next, the first author interviewed with the senior researcher present. The last 16 interviews were conducted by the first author only. The interviewers did not have a private or professional relationship with any of the participants prior to the interviews. Each interview lasted 45–70 min. The participants were free to choose where they wished to be interviewed, either in a private room at the hospital or in their home. To facilitate reflection and conversation, a semi-structured interview guide with follow-up questions was developed based on previous research on risk factors for persistent pain [10, 24] and key topics from the previous longitudinal study from which the patients were recruited [5, 25]. The interview guide contained questions concerning the history of “knee pain prior to the operation”, i.e., the character and severity of the knee pain, and duration before the operation. Questions concerning “events in life” included thoughts on what the participants regarded as meaningful or important things/events in life, as well as any previous physically or psychologically painful life experiences. Follow-up questions gave participants the opportunity to elaborate on issues that were important to them. We pilot-tested the interview guide on three patients, and no changes were considered necessary. We audio-recorded all interviews.

As part of the previous longitudinal study, participants self-reported their number of painful sites through the Brief Pain Inventory (BPI) questionnaire and their chronic illnesses or longlasting sequalae after injuries through the *Socio now pop* (SNP) questionnaire prior to surgery.

### Analysis

A professional transcriber transcribed the interviews verbatim. The first author (VBS) checked and validated the transcripts against the recordings. The data was then analyzed by inductive qualitative content analysis [26, 27]. Two of the authors (VBS, USO) independently read all transcripts to get an overview of the data. Guided by the study aim, they independently identified meaning units containing the stories and expressions of the patients’ earlier painful or stressful life experiences. The authors discussed the meaning units, which were then condensed and coded using descriptions close to the text [26]. The codes were searched for patterns, similarities and differences and sorted into categories. This is a process that involves abstraction and interpretation of the codes, highlighting the unique experiences expressed by patients. Ultimately, the categories were sorted into themes for re-contextualization [26]. The first and second authors (VBS, USO) analyzed the data while three of the co-authors (SAS, AD, MFL) posed critical questions during the analytical process to explore alternative interpretations. Discussion between the authors also ensured that no relevant data had been excluded and that no irrelevant data was included in the analysis [27]. All of the authors agreed on the final themes. The audit trail of the analysis and the findings are shown in Table 1.

**Table 1:** Illustration of the analytical process

Meaning unit	Condensed meaning unit	Code	Category	Theme
I had pain for about ten years, I think. I was limping around 3–4 months of the year. It came in periods. It was very painful (Haakon)	I had pain for about ten years	Pain for years	Long lasting knee pain	Painful years -the burden of living with long lasting pain
I was on 50% disability benefits because of my fibromyalgia (Randi)	I was on disability benefits because of fibromyalgia	Reduced work capability because of widespread pain	The difficult balance of pain and work- and social life	
I had surgery a couple of times on my back too. It has been pretty major operations. And when the knees started to hurt too, I was pretty miserable for a while there (Dagny)	I had surgery a couple of times on my back too. When the knees started to hurt too, I was pretty miserable for a while	Back pain and knee pain	Double burden of pain in multiple locations	
Well, I got rheumatism at the age of 24. It was terribly painful for a long time, coming and going, before it burnt out so to speak. So, I am used to having pain (Signe)	I got rheumatism at the age of 24. It was terribly painful for a long time. So, I am used to having pain	Resolved long lasting painful condition as a young adult	Suffered from long lasting painful condition	
I have been so low in my life because of a very difficult marriage. And it was all about destroying me as the open-hearted person I was. So, it has been a pain so great in my life. But it was impossible for me to leave him, it was dreadful (Ingeborg)	I have been in a very difficult marriage. It was all about destroying me as the open-hearted person. It has been a pain so great	A painful marriage	A life of distress and unhappiness	The burden of living with psychological distress
I was very worried about the analgesia if it would work and such. No, that was very tough. I was afraid and anxious and such (Einar)	I was very worried about the analgesia if it would work and such. I was afraid and anxious and such	Afraid and anxious before surgery	Preoperative anxiety	
My mother, she was old when she had me. She had so much pain, she became all twisted and crippled and she took so much pain medications and became odd from them. In addition: my parents had a bad marriage. I think her pains got worse from it. So, I have seen her, and I just did not want to end up like her (Inger)	My mother had so much pain, she became odd from pain medications, and she had a bad marriage. So I have seen her, I did not want to end up like her	Growing up with a mother in pain	Emotional struggles of difficult family relations	

### Trustworthiness

Credibility was enhanced by recruiting a sample including both sexes and ages ranging from 48 to 84 years at the time of surgery. This contributes to a rich variation of experiences earlier in life. The interview guide enabled participants to communicate, tell stories and emphasize issues that were important to them. Member checking was not carried out, however, during the interviews, follow-up questions were asked for validation and allowed the participants to clear up any misinterpretations of their statements. All participants were asked questions according to the themes in the interview guide, to ensure consistency during data collection. However, the participants’ unique experiences form each individual interview and follow-up questions were tailored to each individual participant, enabling them to elaborate, describe and have additional time to tell their individual story.

The first and second authors are registered nurses with clinical experience on orthopedic wards. Their preconception of patients with persisting pain was that these patients often are vulnerable persons with high levels of stress due to pain. To enhance reflexivity and transparency, these authors’ preconceptions were discussed with the co-authors. Researcher triangulation (VBS, USO, MFL, SAS, AD) was applied to facilitate different perspectives in the analytical process [28]. To enhance dependability, the first author (VBS) received training from a senior qualitative researcher (AD) on how to conduct interviews and a semi-structured interview guide was used. To facilitate the transferability of our findings [29], presentations of the sample, data collection and analytical process and rich descriptions of the findings illustrated with relevant quotes were provided. This was to enable the reader to consider whether the findings are relevant and applicable to their context.

### Ethics

The study was approved by the Regional Committee for Medical and Health Research Ethics in Norway (reference number 2011/1755) and the Data Protection Officer at Lovisenberg Diaconal Hospital. Patients were informed about the study in writing and verbally. All participants signed an informed consent form before the quantitative longitudinal study and another regarding the qualitative interviews. Participants had the right to withdraw from the study at any time until the findings were published. Their confidentiality and anonymity were safeguarded according to local and national regulations.

## Results

Of the 31 eligible patients, two had died, and six patients declined to participate due to illness. The final sample consisted of the remaining 13 women and 10 men, with a median age of 67 (48–84) years at the time of surgery. Prior to surgery, six reported having at least one chronic illness and 16 reported having two or more painful sites. Patient characteristics are shown in Table 2. We have assigned each participant an alias to ensure anonymity. Two themes were identified from the data analysis: painful years - the burden of living with long lasting pain, and the burden of living with psychological distress.

**Table 2:** Overview of participants’ preoperative characteristics

Participant alias	Sex	Cohabitation status	Chronic illness	Number of painful sites
Amund	M	Lives with others	No	2
Astrid	F	Lives with children	No	4
Audun	M	Lives with partner /spouse	No	2
Dagny	F	Lives with partner /spouse	-	2
Ingeborg	F	Lives with partner /spouse	Yes	6
Liv	F	Lives with partner /spouse	-	1
Arne	M	Lives with partner /spouse	No	1
Brynjar	M	Lives alone	No	3
Erlend	M	Lives alone	No	1
Gjermund	M	Lives alone	No	1
Solveig	F	Lives alone	-	3
Inger	F	Lives alone	No	4
Harald	M	Lives with partner /spouse	Yes	5
Signe	F	Lives with partner /spouse	No	1
Ragnhild	F	Lives alone	Yes	5
Sigrid	F	Lives with partner /spouse	No	1
Randi	F	Lives with partner	Yes	3
Idunn	F	Lives with partner /spouse and children	No	4
Olav	M	Lives with partner /spouse	No	1
Sonja	F	Lives with partner /spouse	No	2
Haakon	M	Lives alone	Yes	8
Martha	F	Lives alone	Yes	10
Einar	M	Lives with partner /spouse	No	2

Definitions: - = missing

### Painful years - the burden of living with longlasting pain

#### Long lasting painful conditions

Nearly all the participants experienced living with painful comorbid conditions, such as migraine, back pain, endometriosis, fibromyalgia, and rheumatism, in addition to their longlasting OA knee pain. Of note, some of these were not reported in the preoperative questionnaires (Table 2). Others had additional symptomatic joints such as a painful shoulder, ankle, hip, or contralateral knee. Several had experienced traumatic and painful accidents, such as falls due to ski accidents, motor vehicle accidents or work accidents with trauma to different body parts, that led to hospitalization, and some struggled with sequelae after the accidents. Gjermund experienced long lasting severe pain after a trauma to his hip: “I was up on the roof shoveling snow off the roof, and suddenly I was hanging by one arm from the barge board, and of course I had already cleared the terrace for snow. So, I fell 4 meters down on the terrace on the side. I tore tendons and broke my hip in three places. It was a dreadful pain. That pain in the hip lasted for such a long time, luckily it has become better now.”

Migraine and recurrent headaches were experienced as severe impediments in everyday life and were described as a violent pain leaving them nauseous and unable to function for days at a time. Audun described living with longlasting pain like this: “I’ve had a lot of migraines throughout my life. That has nothing to do with my knee, but it has to do with pain. And that means that I’ve been accustomed, really, to something in my body causing me pain”.

Several female participants suffered from endometriosis, fibromyalgia, and rheumatism, illnesses characterized by multiple painful locations that impaired and restricted them in everyday life. Randi, for example, struggled with endometriosis from adolescence to the age of 40: “Imagine, from I was 15 years old until 40, having all that pain”. She expressed that she was not taken seriously by doctors or at school as a young adult. She felt left to her own devices with longlasting and severe pain that limited her to such a degree that she missed out on social activities and school. She was later diagnosed with fibromyalgia.

Several had experienced severe back pain, described as crippling, leaving them immobilized. Brynjar explained that he had internalized a special walk for several years, bending over, to ease the pain from his spinal stenosis before he finally underwent spinal surgery. These participants continued to compare migraine and back pain to new painful experiences in life, describing them as the worst pain they had ever experienced, and more painful than the knee pain.

#### The double burden of pain in multiple locations

Pain in other joints was also expressed as problematic and exhausting by several participants. Typically, they had endured these other painful conditions for many years, sometimes long before they started to experience osteoarthritic knee pain. These participants emphasized the double burden of having pain in multiple locations. Living with multiple pain locations was described as troubling and stressful. When the knee pain started to manifest itself, these previous pain conditions became especially difficult to endure. As Liv expressed: “You know, I have had a lot of back pain too, I had terrible back pain for many years, but it all got a bit too much for me, when my knees started hurting at the same time ”.

#### The difficult balance of pain and work and social life

Most of the participants had memories of many increasingly painful years, one up to 13 years before surgery. As a result of enduring pain over a long period of time, many expressed in different ways that their QOL was significantly reduced. They found themselves worried about the future, sleepless and distressed. Working and keeping a social life at the same time was described as difficult due to exhausting pain. As a result of debilitating knee pain, many participants were not able to participate fully in their personal or work life. Some prioritized work over having a social life because working was a necessity to feel normal. However, after work they had no energy left, leaving them isolated and excluded from participating in social activities with the important people in their lives. Furthermore, several participants described their sleep quality deteriorating because of pain. Sleepless nights also harmed their QOL and left them tired and unable to participate in activities during the day. Harald described his knee pain as very troublesome for 3–4 years, to such a degree that it disturbed his sleep. He and others described the time with pain before surgery as difficult, tough, or trying and some used metaphors to describe their frustration and exhaustion. Two described their experience of pain like “almost hitting a wall” and another described painful nights as “tossing and turning in bed “ indicating the challenges they faced in dealing with the pain.

#### Physical inactivity

Many participants expressed that they had become physically inactive due to pain. This left them frustrated and feeling out of touch with their normal selves, as typically physically active Norwegians. Amund described his experiences like this: “As long as I had this pain, I could do nothing, I couldn’t bend the knee, I couldn’t walk, so it was a rough time before surgery. This lasted for about two years before I had the knee surgery”.

#### Enduring long lasting pain in addition to OA pain, and then, postsurgical pain

At the time of TKA surgery, some participants (n = 5) no longer had pain in other locations - it had resolved. This was especially the case with some of the participants who endured back pain due to spinal stenosis or disc herniation, and one participant with rheumatic pain conditions that had “burned out” before their knee operation. However, most of the participants still suffered from pain elsewhere, thus they experienced the double burden of OA pain combined with other painful sites. This double burden of pain continued into the postoperative period when postoperative pain and other painful sites interfered with rehabilitation. Despite years of suffering from painful comorbid diseases, many participants displayed a strong mindset and a perseverance. They told their stories with a sense of humor and trying to make sense of their different painful experiences. A recurrent comment among the participants was “I think I’ve a very high tolerance for pain.”

### The burden of living with psychological distress

#### Emotional struggles

Participants described stories of psychologically stressful events, such as the loss of a close relation and grief, as well as difficult marriages, divorces, and family relations. The participants expressed that they had not discussed such experiences with others, and that these experiences had left them with emotional scars. Some of them still struggled emotionally and had yet to process and come to terms with their experiences.

After losing the closest person in their life, patients described stories of grief and mourning, and a feeling of abandonment and lack of control over what happened to them. Gjermund, who had several experiences of the loss of people close to him, both early and later in life, expressed it like this: “Grief work is tough work. I´m still not done with those months from when my wife got sick and until she died. I struggle with that. And that doesn’t make things better.”

Some had sought help to get over their emotional struggles, while others had internalized their emotional distress. It stuck with them, and they never truly got over it, even years after the events. Martha expressed her experience of a difficult break-up like this: “I experienced a divorce that I never truly got over. (…) People around me said I should be done with it, and I took that …well, that went on inside of me, so, I had to seek therapy in the end for that. (…). I think it got stuck in my body because I didn’t breathe properly and such.” Others told stories of difficult and stressful family relations due to parents with painful illnesses and extensive use of pain medications, expressing how this shaped their view on pain medication and pain behavior - they did not wish to become like their parents in this respect.

#### Anxiety and fear

A few participants told stories about a general feeling of anxiety, fear, and reluctance to undergo surgery because of previous experiences with uncontrolled postoperative pain. Erlend described going through an earlier TKA surgery with inadequate pain management. He elaborated: “I was in so much pain that I almost cried. They would only give me paracetamol, so I walked out into the hospital corridor. I remember tossing my crutch because I was so angry and in pain, but they wouldn´t get me anything but paracetamol. That has stuck with me, in my head.” Previous stressful events in life were also described as a reason for feeling anxious and reluctant to undergo surgery. Some postponed surgery because they were concerned about the possibility of something going wrong. Ingeborg had a traumatic experience with the school dentist as a child, which stayed with her into adulthood. She explained: “I’m a sissy when it comes to pain. I don’t know, but when someone else is doing this to you, that’s the worst. When I moved to the city to live on my own, I didn’t dare go to the dentist for two years”.

## Discussion

This qualitative study aimed to explore stories of previous painful or stressful life experiences in a cohort of patients that reported no improvement in pain one year after TKA. We found that most of the patients shared stories of years of struggles with painful comorbid diseases and painful sequelae after accidents prior to surgery, in addition to symptomatic joints. Furthermore, they emphasized life experiences that included loss, grief, and difficult family relations. Thus, their stories also revealed experiences of heavy burdens of psychological distress prior to surgery, unrelated to their painful knee. Furthermore, several expressed a general feeling of anxiety and fear about undergoing new surgeries. We also found inconsistency in what the participants reported through questionnaires, (Table 2) versus their stories of painful conditions during interviews. Those with resolved painful conditions, did not report these, even if the previous longlasting pain had influenced their lives for a long period of time.

Some of our participants had painful symptomatic knees for many years, one even up to 13 years, prior to their TKA surgery. This finding corresponds with one of the strongest independent predictors of PPP, namely preoperative longlasting pain [10, 30]. In addition, participants shared stories of severe pain experienced in relation to accidents. These experiences were highlighted as memories that had stuck with the participants throughout life. Some of the participants continued to struggle with painful sequelae after their accidents. In addition to painful knees, many participants had struggled with other painful conditions for years. Earlier experiences of pain and earlier stressful experiences may lead to a more sensitized pain perception [31–33] although the underlying factors in the complex matter of pain sensitization are still unclear [34].

Several of the female patients in our study were diagnosed with fibromyalgia. Brummett et al. [35] found that fibromyalgia independently predicts poor total knee and hip arthroplasty outcomes, even for patients scoring below the threshold for diagnosing the condition. Three of our participants who underlined the burden of living with rheumatism or other chronic disabilities in the interviews had failed to report these conditions in the preoperative questionnaires (Table 2). If patients are to be identified for possible pain vulnerability prior to surgery, as suggested by Schug and Bruce [9], health personnel may need to ask the patients directly about earlier painful conditions as the patient may not self report these in questionnaires. In the case of our participants, if the conditions had been resolved by the time of surgery, they may have considered the information irrelevant when asked to fill in the questionnaire, or they may not have understood how this information is relevant in a new context. Our participants expressed limitations in physical activities due to pain in two phases: several years prior to TKA surgery due to OA pain and later, due to PPP. Inactivity deteriorated their QOL, leaving them frustrated and feeling out of touch with their physically active lives. Being physically active may also involve an element of expectations of one´s cultural and personal identity which is essential to feeling whole as a person [36, 37] Importantly, physical inactivity is contradictory to the recommendations for optimal treatment of OA by the Osteoarthritis Research Society International (OARSI), which emphasize the combination of non-pharmacological treatments such as exercise, pacing of activities and weight reduction, if needed [1].

Our findings suggest that pain interfered greatly with participants’ social life and psychological wellbeing. In particular, participants still working expressed difficulties prioritizing between work, or social and family life due of the exhausting pain. A systematic review indicates that patients with chronic pain who wait more than six months for treatment experience increased physical and psychosocial problems, deterioration in HRQOL and increased depression scores [38]. As a result, pain may lead to social isolation, and for younger patients, difficulties working, which can also lead to depression and low self-esteem [39] Furthermore, several of our participants experienced poor sleep quality due to pain. Studies have raised the issue of the bidirectional relationship between pain and sleep [40]. In line with a previous study [41], our participants with excessive preoperative pain seemed to experience a cycle of pain and deteriorating health. In addition, poor preoperative sleep quality has been shown to have a negative impact on PPP [42].

**Fig. 1:**
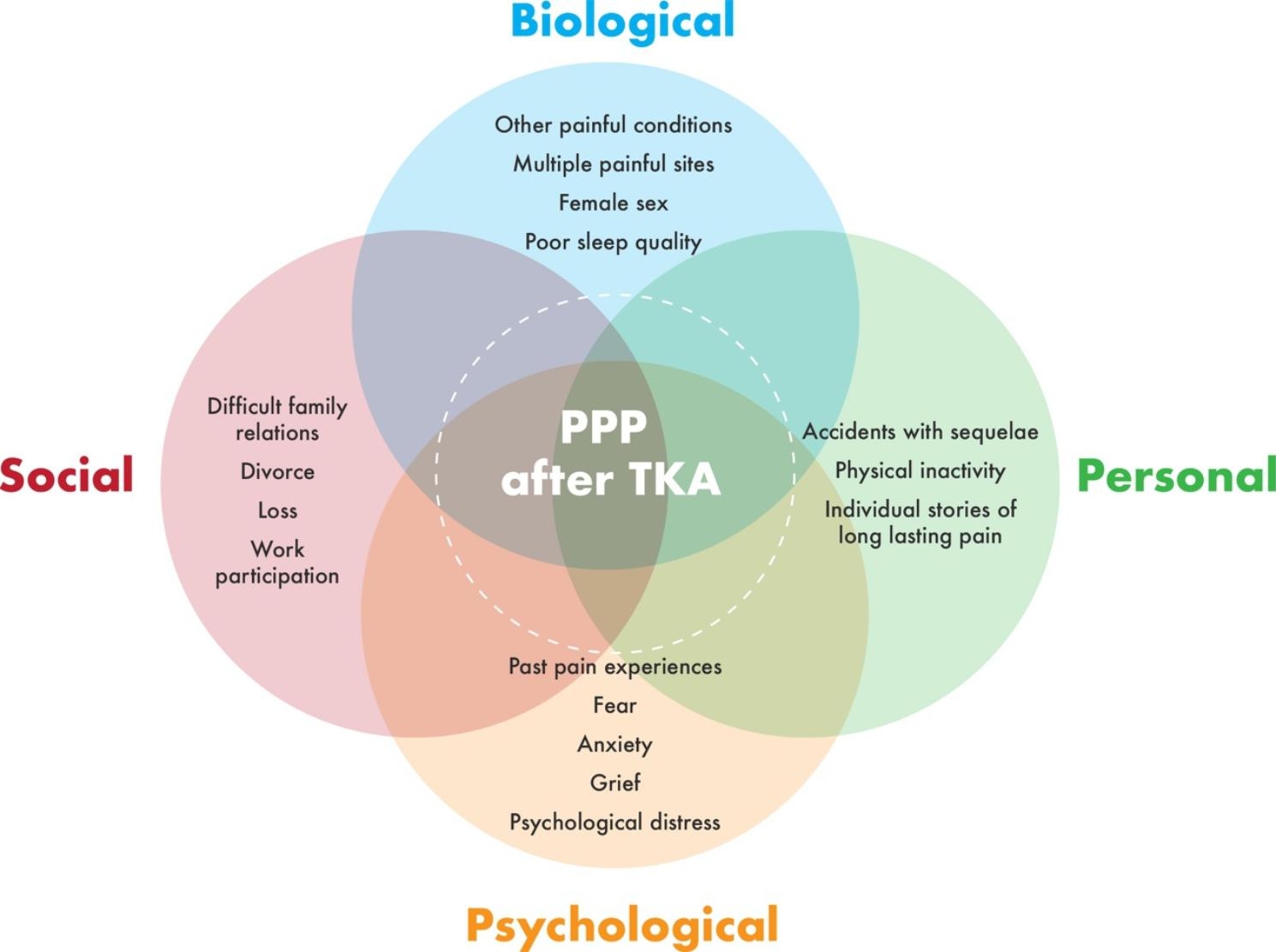
Illustrates the elements in painful experiences from a biopsychosocial pain perspective

Participants shared stories and expressed that they still struggled with loss, grief, difficult family relationships and distress. Some said they had not shared their stories of psychological hardship with others before, leaving them alone with their thoughts and struggles. A systematic review and a meta-analysis highlight the poor outcome after TKA for patients who struggle with psychological health and distress, i.e. depression and anxiety [11, 43]. Psychological stress is a known risk factor associated with postsurgical persistent pain after TKA [10, 30, 43]. We found that these additional psychological stress experiences added to the burdens already carried by our participants. The double burden, thus, is expressed by multiple layers of struggles visualized in both biological and psychological and social components [44] (Fig. 1). Holding stressful experiences in life can complicate the postoperative pain experience and leave individuals more vulnerable to the perception of pain [33]. When considering our findings from a biopsychosocial viewpoint, it is important to consider the passage of time and any experiences that have had a lasting impact in regard to pain and persistent pain.

Some of our participants described a general feeling of anxiety and reluctance to undergo surgery. Some even postponed their surgery due to fear of complications or expectations of severe postoperative pain. Some expressed a sense of helplessness in their painful situation; they no longer felt they could do something to relieve the pain, they felt like they had hit a wall, and they felt exhausted, worried, and distressed. High levels of anxiety may be a reason some patients are more attentive to pain [33]. These descriptions may indicate that some participants tend to catastrophize pain. Pain catastrophizing is defined as an expectation or worry about major negative consequences during an actual or anticipated painful experience. Magnification and helplessness are among the components described [45]. A recent meta-analysis concludes that OA patients should be screened for anxiety and depression for better pain management and to increase clinical awareness around the association between psychological aspects and persistent pain [46]. However, eligibility for surgery should not be influenced by preoperative anxiety or depression symptoms [47, 48]. In addition, OARSI guidelines suggest that patients’ clinical status may improve before surgery if they are contacted regularly by phone [1]. Importantly, patients with PPP have reported a feeling of abandonment post-operatively, expressing a need for more support after TKA [49]. A tailored supplement to treatment such as internet delivered follow-up may also be a way to improve outcomes for vulnerable patients [50].

The first and second authors’ preconception of patients with persistent pain was that these patients are often vulnerable with high levels of stress. However, our participants showed perseverance and a strong mindset. This was unexpected. Participants sometimes had a humorous twist or tone when telling their stories of painful or stressful experiences. Having in mind that these patients may be reluctant to share their experiences with healthcare personnel [51], humor may be a way of modifying and presenting their stories in a more listener-friendly way.

The perception of pain may be influenced by physical health problems, such as painful conditions and multiple painful sites, one’s psychological state, such as fear, expectations, earlier pain history, family relations and work-life [33]. Thus, several factors are at play and must be considered when exploring the complexity of persistent pain after TKA. The biopsychosocial model [39, 44, 52] is a useful theoretical model for understanding and explaining what our participants highlighted as important memories and experiences, in this context of pain, before their TKA surgery. The model illustrates the complexity of elements that can influence the perception of pain in patients.

### Strengths and limitations

Our study employed purposive sampling of participants from a larger longitudinal quantitative study, which allowed us to conduct this follow-up study targeting an already identified subgroup of patients who did not improve one year after TKA. However, in this follow-up study the participants were interviewed only at one time-point 5–7 years postoperatively as part of a 5 year follow-up. Stories from years prior to surgery may lead to recall bias. However, we aimed to investigate the participants’ own perception of painful stories and what was important and meaningful to them. We believe that the real-life issues and struggles important to the participants were highlighted through their sharing of stories of earlier incidents in life. What participants remember is influenced by time and by the painful experience itself [53, 54]. However, we believe that we achieved what we aimed to; namely explore earlier painful and stressful experiences considered important and meaningful to the participants in the context of experiencing pain [23]. When discussing the findings within a biopsychosocial perspective, the passing of time and highlighting experiences that have stuck with them, is in fact highly relevant in the context of pain perception and persistent pain [33, 54].

Having data from questionnaires and interviews allowed us to detect inconsistency in what patients report on painful conditions. We found that patients excluded information on painful accidents with sequelea and painful comorbidity in questionnaires while elaborating on them in interviews. The theoretical model of information power was employed to evaluate sample size [55]. The sample size generated sufficient information power as the participants´ characteristics were highly specific for the aim. The interview dialogue and setting enabled the participants to share their stories undisturbed in a safe and quiet environment, consequently, generating rich data [8, 55]. The participants are all from one single orthopedic unit in Norway. The unit is however one with the highest volume of TKA surgery in Norway, allowing for a subgroup of non-improvers of a certain number.

## Conclusion

Participants in this study told stories of lives with emotional struggles and long lasting painful conditions. The double burden of more painful conditions often in addition to psychological stress left the participants struggling, often years before surgery. Their stories described a vicious cycle of pain and deteriorating QOL, physically, socially, and psychologically prior to TKA surgery.

## Implications for clinical practice and further research

The study highlights the importance of considering patients’ preoperative stories of pain, as well as their psychological and social struggles, for better identification of those at risk of PPP. Nurses, physiotherapists, and physicians may tailor healthcare for these patients if their challenges are identified preoperatively. Identifying and assessing the challenges enables personalized care and support, such as advice on pain management, cognitive support, guided rehabilitation and coping strategies both pre- and post-surgery. Addressing patients´ high levels of pain and related or unrelated psychological struggles before surgery can thus improve outcomes and reduce the risk of PPP.

The present study provides insight into the non-improver TKA patients as individuals and offers new approaches for identifying those patients who will benefit from individualized perioperative pain management. These findings could be subject to further quantitative investigations.

## Data Availability

The datasets generated and analyzed during the current study are not publicly available due to not compromise individual privacy and legal restrictions. A minimal dataset may however be available from the authors upon reasonable request and with permission from the Regional Committee in Ethics in Medical Research in South-East Norway (REC) and the hospitals’ Data Protection Officers.

## Abbreviations

BPI: Brief Pain Inventory
HRQOL: Health Related Quality of Life
OA: osteoarthritis
OARSI: the Osteoarthritis Research Society International
PPP: Persistent post-surgical Pain
TKA: Total Knee Arthoplasty
